# Four new species of *Phyllosticta* from China based on morphological and phylogenetic characterization

**DOI:** 10.1080/21501203.2023.2225552

**Published:** 2023-07-04

**Authors:** Xiao-Nan Sui, Mei-Jun Guo, Hao Zhou, Cheng-Lin Hou

**Affiliations:** College of Life Science, Capital Normal University, Beijing, China

**Keywords:** multigene phylogeny, new species, taxonomy

## Abstract

*Phyllosticta* (*Phyllostictaceae, Botryosphaeriales*) species are widely distributed globally and constitute a diverse group of pathogenic and endophytic fungi associated with a broad range of plant hosts. In this study, four new species of *Phyllosticta*, i.e. *P. endophytica, P. jiangxiensis, P. machili*, and *P. xinyuensis*, were described using morphological characteristics and multi-locus phylogeny based on the internal transcribed spacer region (ITS) with intervening 5.8S rRNA gene, large subunit of rRNA gene (nrLSU), translation elongation factor 1-alpha gene (*tef1*), actin gene (*act*), and glyceraldehyde-3-phosphate dehydrogenase gene (*gapdh*). *Phyllosticta machili* is the first species of this genus reported to infect plants of the *Machilus* genus.

## 1. Introduction

The genus *Phyllosticta* (*Phyllostictaceae, Botryosphaeriales*, and *Ascomycota*) was established by Persoon (Persoon 1818). Presently, a total of 3,215 names are documented for *Phyllosticta* in the Index Fungorum (accessed on 29 March 2023). However, many of these names have been synonymised. Currently, 1,499 species are recognised in this genus (Bánki et al. 2022). Most members of *Phyllosticta* are important plant pathogens and endophytes with a wide host range and geographical distribution (Van der Aa and Vanev 2002). Some *Phyllosticta* species cause significant impacts on economic crops, such as citrus black spot caused by *P. citricarpa* (McAlpine) Aa, which is regarded as a quarantine pathogen in Europe and the USA (Baayen et al. 2002; Wulandari et al. 2009; Glienke et al. 2011), black rot disease on grapevines by *P. ampelicida* (Engelm.) Aa species complex (Wicht et al. 2012), and banana freckle disease by *P. musarum* (Cooke) Aa species complex (Pu et al. 2008; Wong et al. 2012). In addition, some species of *Phyllosticta* have also been isolated from a wide range of hosts as endophytic fungi. For example, *P. capitalensis* Henn. is an endophytic fungus distributed in more than 20 different families of plants in eight countries (Baayen et al. 2002; Wulandari et al. 2010; Glienke et al. 2011). However, endophytes can sometimes be transformed into pathogens, such as *P. capitalensis*, which can cause black freckle disease on *Rubus chingii* HU and leaf spots on *Ligustrum japonicum* Thunb. as a pathogen, thus affecting the visual value of ornamental plants (Sabahi et al. 2022; Zhang et al. 2022). Some species of *Phyllosticta* can survive by degrading organic material from dead plants as saprobes, such as *P. carpogena* (Shear) Aa and *P. ericae* Allesch (Trigiano et al. 2004) occurring on *Rubus* sp. (*Rosaceae*) and *Erica carnea* (*Ericaceae*), respectively (Van der Aa and Vanev 2002).

In China, some species of *Phyllosticta* can infect economic plants and cause economic losses. For example, *P. theicola* Curzi and *P. capitalensis* can both cause leaf spots on *Camellia sinensis* (L.) O. Ktze. (Cheng et al. 2019; Tian et al. 2019). *Phyllosticta capitalensis* can cause leaf spots on oil palms (Nasehi et al. 2020), and *P. citricarpa* can cause black spot disease on citrus leading to black spots on fruit and premature fruit drop (Wang et al. 2012; Tran et al. 2020).

The aim of this study was to identify *Phyllosticta* strains isolated from different host plants in China by morphological characterisation and multi-gene phylogenetic analysis.

## 2. Material and methods

### 2.1. Isolation and culture

Diseased leaf tissues and healthy tissues from different hosts were collected from China’s Anhui, Hubei, Yunnan, and Jiangxi provinces. Fragments (5 mm × 5 mm) were taken from the margin of leaf lesions and healthy parts of leaves. All fragments were disinfected in 75% ethanol for 30 s, followed by 10% sodium hypochlorite for 3 min, washed in sterile water three times, then placed on potato dextrose agar (PDA), and cultured at room temperature (25 ± 3) °C. Cultures were dried after sporulation and sent as holotype to Chinese Academy of Forestry (CAF; http://museum.caf.ac.cn), and ex-type living cultures of new species were deposited in the China Forestry Culture Collection Center (CFCC; http://cfcc.caf.ac.cn/) and Capital Normal University Culture Collection Center (CNUCC).

### 2.2. Morphological analysis

Isolates were cultured for 12–15 days and examined periodically until sporulation. The pycnidia and conidia on cultures were examined with a dissecting microscope (Nikon SMZ-1000) and an upright microscope (Olympus BX51). Mature pycnidia were immersed and crushed in water, releasing both conidia and conidiogenous cells on glass slides. Twenty of each structure, including pycnidia, conidiogenous cells, and conidia, were measured to calculate the average size as described. Colony colours were described based on the ColorHexa code (https://www.colorhexa.com/).

### 2.3. Phylogenetic analysis

Isolates were cultured on PDA for seven days at room temperature. Mycelia were collected with a sterile inoculation shovel, and genomic DNA was extracted using the M5 Plant Genomic DNA Kit (Mei5 Biotechnology Co., Ltd., China) following the manufacturer’s instructions. Five loci, including ITS, *act*, nrLSU, *gapdh*, and *tef1* (Table 1), were amplified and sequenced using primers pairs ITS-1F (Gardes and Bruns 1993) and ITS-4 (White et al. 1990), ACT-512-F and ACT-783-R (Carbone and Kohn 1999), LR0R (Moncalvo et al. 1995) and LR5 (Vilgalys and Hester 1990), Gpd1-LM and Gpd2-LM (Myllys et al. 2002), and EF1-728F and EF2 (Carbone and Kohn 1999), respectively (Table 1). The 25 μL reaction volume consisted of 12.5 μL Master Mix (Mei5 Biotechnology, Co., Ltd., Beijing, China), 1 μL of each forward and reverse primer, 1 μL template genomic DNA (about 10 ng/μL), and 8.5 μL distilled deionised water. PCR products were visually inspected in agarose electrophoresis gels and compared with band intensities of 200 bp DNA ladders.

**Table 1.** Genes, PCR primers, procedures, and references used in this study.

Genes	Primers	Procedures	Reference
ITS	ITSIF/ITS4	95 °C: 4 min; (95 °C: 30 s, 55 °C: 30 s, 72 °C: 45 s) × 35 cycles; 72 °C: 10 min	Gardes and Bruns 1993
*act*	ACT-512-F/ACT-783-R	95 °C: 4 min; (95 °C: 30 s, 58 °C: 30 s, 72 °C: 45 s) × 35 cycles; 72 °C: 10 min	Carbone and Kohn 1999
*gapdh*	Gpd1-LM/Gpd2-LM	95 °C: 4 min; (95 °C: 30 s, 60 °C: 30 s, 72 °C: 45 s) × 35 cycles; 72 °C: 10 min	Myllys et al. 2002
nrLSU	LR0R/LR5	95 °C: 4 min; (95 °C: 30 s, 51 °C: 30 s, 7 2 °C: 45 s) × 35 cycles; 72 °C: 10 min	Vilgalys and Hester 1990
*tef1*	EF1-728F/EF2	95 °C: 4 min; (95 °C: 30 s, 48 °C: 30 s, 72 °C: 45 s) × 35 cycles; 72 °C: 10 min	Carbone and Kohn 1999

Purification and sequencing were performed by Zhongkexilin Biotechnology Company (Beijing, China). DNA sequences using forward and reverse primers were aligned using Editseq v5.0 to obtain consensus sequences. The new sequences in this study were submitted to the GenBank. Other sequences of the ITS, nrLSU, *tef1, act*, and *gapdh* genes were downloaded from GenBank (Table 2). The segmentation homogeneity test was performed to determine the consistency of gene segments (Farris et al. 1994; Huelsenbeck et al. 2003). Aligned using the online MAFFT tool (https://www.ebi.ac.uk/Tools/msa/mafft/) and edited using Gblocks (https://www.phylogeny.fr/one_task.cgi?task_type = gblocks) by selecting DNA and all options for less stringent criteria.Strain names and sequences of new species in this study are indicated in bold. Isolates marked with “*” are ex-type or ex-epitype strains.

**Table 2.** Isolates and GenBank accession numbers used in the phylogenetic analyses of *Phyllosticta.*

Species	Isolate	Host	Country	ITS	nrLSU	*tef1*	*act*	*gapdh*
*Botryosphaeria obtusa*	CMW8232	*Conifers*	South Africa	AY972105	–	DQ280419	AY972111	–
*Guignardia gaultheriae*	CBS 447.70*	*Gaultheria humifusa*	USA	JN692543	KF206298	JN692531	KF289248	JN692508
*Phyllosticta abieticola*	CBS112067*	*Abies concolor*	Canada	KF170306	EU754193	–	KF289238	–
*P. alliacea*	MUCC0014*	*Aloe ferox*	South Africa	AB454263	–	–	AB704207	–
*P. ampelicida*	ATCC 200578*	*Vitis riparia*	USA	KC193586	–	–	KC193581	KC193584
*P. ardisiicola*	MUCC0031*	*Ardisia crenata*	Japan	AB454274	–	–	AB704216	–
*P. aspidistricola*	MUCC0010*	*Aspidistra elatior*	Japan	AB454260	–	–	AB704204	–
*P. austroafricana*	CPC 31920 = CBS 144593*	Leaf spots of unidentified deciduous tree host	South Africa	MK442613	MK442549	MK442704	MK442640	–
*P. beaumarisii*	CBS 535.87*	*Muehlenbeckia adpressa*	Australia	NR145235	NG058040	KF289170	KF306232	KF289074
*P. bifrenariae*	CBS 128855*	*Bifrenaria harrisoniae*	Brazil	JF343565	KF206209	JF343586	JF343649	JF343744
*P. camelliae*	MUCC0059	*Camellia japonica*	Japan	AB454290	–	–	AB704223	–
*P. capitalensis*	IMI260.576*	*Mangifera indica*	India	JF261459	KF206222	JF261501	JF343641	JF343748
*P. capitalensis (Guignardia mangiferae)*	CPC 18848*	*Stanhopea graveolens*	Brazil	JF261465	KF206255	JF261507	KF289289	JF343776
*P. carissicola*	CPC25665*	*Carissa macrocarpa*	South Africa	KT950849	KT950863	KT950879	KT950872	KT950876
*P. cavendishii*	BRIP 55419*	*Musa* sp. cv. Formosana (AAA)	Taiwan	JQ743562	–	KF009743	KF014080	–
*P. citriasiana*	CBS 120486*	*Citrus maxima*	Thailand	FJ538360	KF206314	FJ538418	FJ538476	JF343686
*P. citribraziliensis*	CBS 100098*	*Citrus* sp.	Brazil	FJ538352	KF206221	FJ538410	FJ538468	JF343691
*P. citricarpa*	CBS 127454*	*Citrus sinensis*	Australia	JF343583	KF206306	JF343604	JF343667	JF343771
*P. citrichinaensis*	ZJUCC 200956*	*Citrus reticulata*	China	JN791620	–	JN791459	JN791533	–
*P. citrimaxima*	CPC 20276*	*Citrus maxima*	Thailand	KF170304	KF206229	KF289222	KF289300	KF289157
*P. concentrica*	CPC18842	*Hedera* sp.	Spain	NR153218	NG069154	KF289228	KF289288	KF289163
*P. cordylinophila*	CPC 20261*	*Cordyline fruticosa*	Thailand	KF170287	KF206242	KF289172	KF289295	KF289076
*P. cussonia*	CPC 14875*	*Cussonia* sp.	South Africa	JF343579	KF206278	JF343600	JF343663	JF343765
*P. cryptomeriae*	MUCC 0028*	*Cryptomeria japonica*	Japan	NR172812	–	–	AB704213	–
*P. dendrobii*	CGMCC 3.18666*	*Dendrobium nobile*	China	MF180193	MF180210	MF180202	MF180220	MF180229
*P. doitungensis*	MFLU 21–0175*	*Dasymaschalon* sp.	Thailand	OK661033	OK661034	–	–	–
*P. encephalarticola*	CPC 35970*	*Encephalartos* sp.	South Africa	NR166311	NG068314	–	–	–
***P. endophyticum***	**S9615-1**	***Cunninghamia lanceolata***	**China**	**OQ996246**	**OQ996159**	**OQ995169**	**OQ995154**	**OQ995165**
***P. endophytica***	S317B-1*	***Cunninghamia lanceolata***	**China**	**OQ996245**	**OQ996158**	**OQ995174**	**OQ995153**	**OQ995160**
***P. endophytica***	**S374-1**	***Cunninghamia lanceolata***	**China**	**OQ996250**	**OQ996157**	**OQ995173**	**OQ995152**	**OQ995161**
*P. ericarum*	CBS 19744*	*Erica gracilis*	South Africa	KF206170	KF206253	KF289227	KF289291	KF289162
*P. fallopiae*	MUCC0113*	*Fallopia japonica*	Japan	AB454307	–	–	AB704228	–
*P. foliorum*	CBS 447.68*	*Taxus baccata*	Netherlands	KF170309	KF206287	KF289201	KF289247	KF289132
*P. gwangjuensis*	CNUFC NJ-1-12*	*Torreya nucifera*	Korea	OK285195	–	OM038511	OM001471	–
*P. hagahagaensis*	CPC 32799 = CBS 144592*	*Carissa bispinosa*	South Africa	MK442614	MK442550	MK442705	MK442641	MK442657
*P. hostae*	CGMCC 3.14355*	*Hosta plantaginea*	China	JN692535	–	JN692524	JN692512	JN692503
*P. hubeiensis*	CGMCC 3.14986*	*Viburnum odoratissimim*	China	JX025037	–	JX025042	JX025032	JX025027
*P. hymenocallidicola*	CBS 131309*	*Hymenocallis littoralis*	Australia	JQ044423	JQ044443	KF289211	KF289242	KF289142
*P. hypoglossi*	CBS 434.92*	*Ruscus aculeatus*	Italy	FJ538367	KF206299	FJ538425	FJ538483	JF343695
*P. ilicis-aquifolii*	CGMCC 3.14358*	*Ilex aquifolium*	China	JN692538	–	JN692526	JN692514	–
*P. illicii*	CGMCC 3.18670*	*Illicium verum*	China	MF180195	MF180212	MF180203	MF180221	–
***P. jiangxiensis***	S686-1*	***Camellia oleifera***	**China**	**OQ996247**	**OQ996160**	**OQ995170**	**OQ995149**	**OQ995163**
***P. jiangxiensis***	**S687-1**	***Camellia oleifera***	**China**	**OQ996252**	**OQ996164**	**OQ995176**	**OQ995156**	**OQ995164**
***P. jiangxiensis***	**S688-1**	***Camellia oleifera***	**China**	**OQ996253**	**OQ996165**	**OQ995177**	**OQ995157**	**OQ995167**
*P. kerriae*	MUCC0017*	*Kerria japonica*	Japan	AB454266	–	–	AB704209	–
*P. leucothoicola*	MUCC0553*	*Leucothoe catesbaei*	Japan	AB454370	–	–	KF289310	–
*P. ligustricola*	MUCC0024*	*Ligustrum obtusifolium*	Japan	AB454269	–	–	AB704212	–
***P. machili***	S661-1*	***Machilus pauhoi***	**China**	**OQ996249**	**OQ996162**	**OQ995172**	**OQ995151**	**OQ995159**
***P. machili***	**S662-1**	***Machilus pauhoi***	**China**	**OQ996254**	**OQ996166**	**OQ995178**	**OQ995158**	**OQ995168**
*P. maculata*	CPC18347*	*Musa* sp. cv. Goly-goly pot-pot (ABB)	Australia	NR147336	NG059472	–	–	–
*P. mangifera-indica*	CPC 20264*	*Mangifera indica*	Thailand	KF170305	KF206240	KF289190	KF289296	KF289121
*P. minima*	CBS 585.84*	*Acer rubrum*	USA	KF206176	KF206286	KF289204	KF289249	KF289135
*P. musicola*	CBS123405*	*Musa acuminata*	Thailand	FJ538334	–	FJ538392	FJ538450	–
*P. neopyrolae*	MUCC0125*	*Pyrola asarifolia*	Japan	AB454318	NG070025	–	AB704233	–
*P. oblongifolae*	SAUCC210052*	*Garcinia oblongifolia*	China	OM248445	OM232088	OM273893	OM273897	OM273901
*P. ophiopogonis*	LrLF11	*Lycoris radiata*	China	MG543713	–	–	–	–
*P. owaniana*	CBS 776.97*	*Brabejum stellatifolium*	South Africa	NR147324	KF206293	FJ538426	KF289254	JF343767
*P. pachysandricola*	MUCC0124*	*Pachysandra terminalis*	Japan	AB454317	–	–	AB704232	–
*P. parthenocissi*	CBS111645*	*Parthenocissus quinquefolia*	USA	EU683672	–	JN692530	JN692518	–
*P. paxistimae*	CBS 112527*	*Paxistima mysinites*	USA	KF206172	KF206320	KF289209	KF289239	KF289140
*P. podocarpi*	CBS 111647	*Podocarpus macrophyllus* var. maki	South Africa	KF766217	–	KF766434	–	–
*P. podocarpicola*	CBS 728.79*	*Podocarpus maki*	USA	KF206173	KF206295	KF289203	KF289252	KF289134
*P. pterospermi*	SAUCC210104*	*Pterospermum heterophyllum*	China	OM249954	OM249956	OM273902	OM273904	OM273906
*P. pterospermi*	SAUCC210106	*Pterospermum heterophyllum*	China	OM249955	OM249957	OM273903	OM273905	OM273907
*P. rhaphiolepidis*	MUCC0432*	*Rhaphiolepis indica*	Japan	AB454349	–	–	AB704242	–
*P. rubra*	CBS 111635*	*Acer rubrum*	USA	KF206171	EU754194	KF289198	KF289233	KF289129
*P. schimae*	CGMCC 3.14354*	*Schima superba*	China	JN692534	–	JN692522	JN692510	JN692506
*P. schimicola*	CGMCC 3.17319*	*Schima superba*	China	KJ847426	–	KJ847448	KJ847434	KJ854895
*P. styracicola*	CGMCC 3.14985*	*Styrax gradiflorus*	China	NR153207	–	JX025045	JX025036	–
*P. telopeae*	CBS 777.97*	*Telopea speciosissima*	Tasmania	KF206205	KF766384	KF289210	KF289255	KF289141
*P. vaccinii*	ATCC 46255*	*Vaccinium macrocarpon*	USA	NR147339	–	KC193582	KC193580	KC193583
*P. vacciniicola*	CPC18590*	*Vaccinium macrocarpum*	USA	–	KF206257	KF289229	KF289287	KF289165
*P. vitis-rotundifoliae*	CGMCC 3.17322*	*Vitis rotundifolia*	USA	KJ847428	–	KJ847450	KJ847436	KJ847442
***P. xinyuensis***	S668-1*	***Camellia oleifera***	**China**	**OQ996248**	**OQ996161**	**OQ995171**	**OQ995150**	**OQ995162**
***P. xinyuensis***	**S669-1**	***Camellia oleifera***	**China**	**OQ996251**	**OQ996163**	**OQ995175**	**OQ995155**	**OQ995166**
*P. aucubae-japonicae*	MAFF 236703*	*Aucuba japonica*	Japan	NR146248	–	KR233310	KR233305	–
*P. gardeniicola*	MUCC0117	*Gardenia jasminoides*	Japan	AB454310	–	–	AB704230	–
*P. iridigena*	CBS 143410*	*Iris* sp.	South Africa	NR161057	–	MG934502	MG934466	–
*P. paracitricarpa*	CBS 141357*	*Citrus limon*	Greece	NR153304	NG069458	–	–	–
*P. speewahensis*	BRIP 58044*	*Vanda* sp.	Australia	NR147342	–	KF017268	–	–
*P. spinarum*	MUCC 2918	*Thujopsis dolabrata*	Japan	LC542612	LC543438	LC543459	LC543478	–

Strain names and sequences of new species in this study are indicated in bold. Isolates marked with “*” are ex-type or ex-epitype strains.

The phylogenetic analyses were based on a combined ITS, nrLSU, *act, tef1*, and *gapdh* matrix, including the type and representative species of *Phyllosticta* (Norphanphoun et al. 2020). A maximum parsimony (MP) tree was constructed with PAUP v4.0b10 (Swofford 2003). Trees were inferred using the heuristic search option with TBR branch swapping and 1,000 random sequence additions. Branches of zero length were collapsed and all equally most parsimonious trees were saved. Tree length (TL), consistency index (CI), retention index (RI), and rescaled consistency index (RC) were calculated for the generated parsimony trees. For the Bayesian inference (BI) analysis, MrModeltest v2.3 with the Akaike information criterion was used as a substitution model for the concatenated dataset (Nylander 2004). The GTR+I+G model was proposed for ITS, nrLSU, and *gapdh*, HKY+I+G for *act*, and GTR+G for *tef1*. 1,000,000 generations were launched with a random starting tree, and Markov chains were sampled every 100 generations. The analysis was stopped when the average standard deviation of the separation frequency was 0.01. During the burn-in phase of the analysis, the first 25% of trees were discarded, and the remaining trees were used to calculate the posterior probabilities (PPs).

## 3. Results

### 3.1. Phylogenetic analysis

Single-gene phylogenetic trees were constructed before the multi-gene phylogenetic analysis to examine topology and clade support based on the ITS, nrLSU, *act, tef1*, and *gapdh* genes (Supplementary Figures 1, 2, 3, 4, and 5). The topology of the single-gene phylogenetic tree for ITS, *act*, and *tef1* was similar to the multi-gene phylogenetic tree, but the support values of the deep nodes were lower. The topology of the single-gene phylogenetic tree of nrLSU and *gapdh* differed slightly from the multi-gene phylogenetic tree.

In the multi-locus phylogenetic analysis, 311 sequences of 81 isolates were used to construct a five-locus phylogenetic tree; *Botryosphaeria obtusa* (Schwein.) Shoemaker (CMW 8232) was used as the outgroup. The dataset of five loci comprised 2,470 characters, including the alignment gaps, of which 623 characters were parsimony-informative, 305 parsimony-uninformative, and 1,542 constants. The analytical analysis yielded 1,000 similar trees, one of which (TL = 3,034, CI = 0.467, RI = 0.727, RC = 0.340, and HI = 0.533) is shown in Figure 1. The Bayesian tree confirmed the topology of the obtained tree using the maximum parsing method.

**Figure 1.**
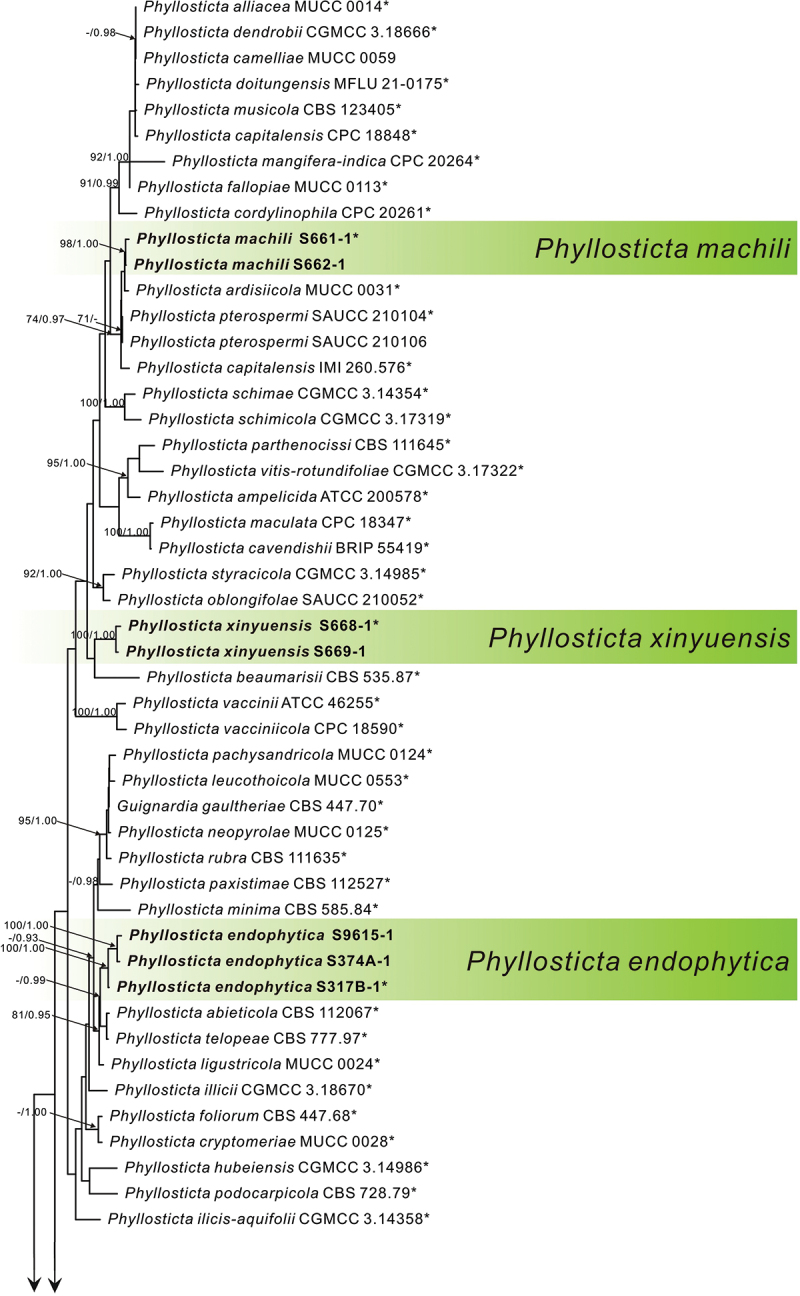
MP phylogenetic tree inferred from combined ITS, *act*, nrLSU, *gapdh*, and *tef1* sequence data from 81 strains of the genus *Phyllosticta* and *Botryosphaeria obtusa* CMW 8232 as an outgroup. MP bootstrap values above 70% and Bayesian PPs above 0.90 are given at the nodes in this order. Strain names and sequences of new species in this study are indicated in bold. Isolates marked with “*” are ex-type or ex-epitype strains.

In phylogenetic analysis, three strains of *P. endophytica* formed a sister clade (MPB = 100, PP = 1.00) with *P. abieticola* Wikee & Crous, *P. telopeae* H.Y. Yip, and *P. ligustricola* Motohashi & C. Nakash. Three strains of *P. jiangxiensis* formed an independent clade with high support (MPB = 100, PP = 1.00). Two strains of *P. machili* clustered with *Phyllosticta ardisiicola* Motohashi, I. Araki & C. Nakash., *P. pterospermi* Z.X. Zhang, X.Y. Liu, Z. Meng & X.G. Zhang, and *P. capitalensis* with high support (MPB = 98, PP = 1.00). Two strains of *P. xinyuensis* clustered with *P. beaumarisii* with high support (MPB = 100, PP = 1.00).

### 3.2. Taxonomy

Based on the phylogenetic analysis and morphological comparisons, 10 strains were attributed to four new species of *Phyllosticta*.

**Figure 2.**
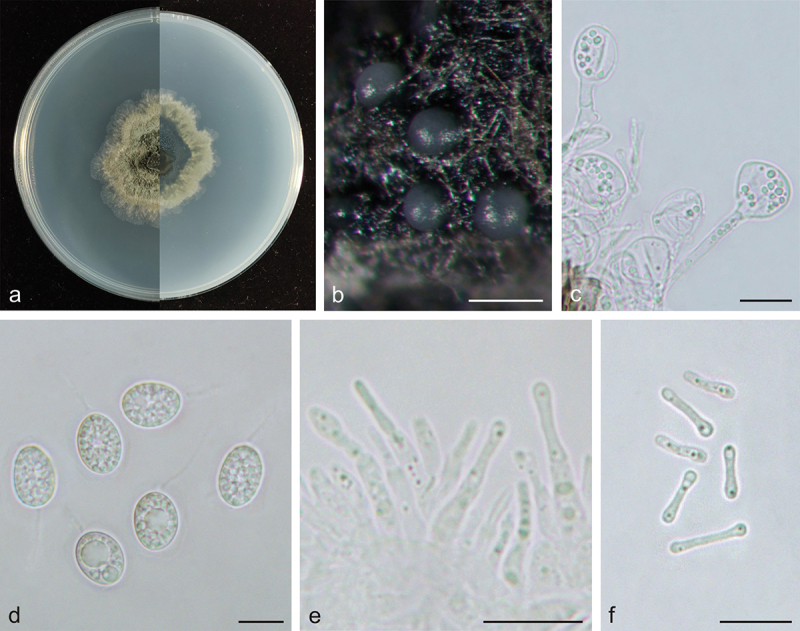
*Phyllosticta endophytica* (holotype, CAF 8000201). a: Colonies (left-above, right-reverse) after 7 days on PDA; b: Conidiomata; c: Conidiogenous cells; d: Conidia; e: Spermatogenous cells producing spermatia; f: Spermatia. Scale bars: 200 μm (b); 10 μm (c–f).

***Phyllosticta endophytica*** X.N. Sui & C.L. Hou, sp. nov. Figure 2

*MycoBank*: 848949.

 *Etymology*: *endophytica*, referring to its lifestyle, endophytic.

 *Type:* China, Yunnan Province: Daguan County Hekou Village, on healthy leaves of *Cunninghamia lanceolata* (Lamb.) Hook., 13 April 2019, Q.T. Wang & C.L. Hou (holotype, CAF 8000201, ex-holotype living culture CNUCC S317-1 = CFCC 59082).

 *Description*: Conidiophores are cylindrical to ampulliform, often reduced to conidiogenous cells. Conidiogenous cells 14.5–28.0 × 2.0–5.0 μm, hyaline, cylindrical. Conidia 12.5–16.0 × 9.0–11.0 μm, mean ± SD = 14.1 ± 0.8 × 10.3 ± 0.6 μm, hyaline, aseptate, ovoid, ellipsoidal to subglobose, surrounded by a thin mucoid sheath, bearing a hyaline, apical mucoid appendages, 7.0–15.0 μm, unbranched, and flexible. Spermatiogenous cells are subcylindrical to ampulliform, 7.0–12.0 × 2.0–3.0 μm. Spermatia aseptate, dumbbell-shaped, 6.0–11.0 × 1.0–2.0 μm, mean ± SD = 8.5 ± 1.4 × 1.5 ± 0.2 μm.

 *Culture characteristics*: Colonies up to 4.3 cm in diameter at 7 days on PDA. Irregular at the edge, dark desaturated green (#273517) to greyish-white (#e0e0e0) on the obverse, and dark lime green (#002700) to greyish-white (#e0e0e0) on the reverse side. Conidiomata are visible after 15 days with dark grey oozing (#a3a3a3) tendrils.

 *Additional specimens examined*: China, Hubei Province: Xiangjia Mountain Villa Tea plantation, on healthy leaves of *Cunninghamia lanceolata*, 6 May 2019, Q.T. Wang & C.L. Hou, living culture S374A-1. China, Anhui Province: Shucheng Hepeng *Camellia* Nursery tea plantation, on healthy leaves of *Cunninghamia lanceolata*, 17 February 2017, Q.T. Wang & C.L. Hou, living culture S9615-1.

 *Notes*: In the phylogenetic analysis, the three strains of *P. endophytica* clustered together with high support values (MPB = 100, PP = 1.00, Figure 1) and formed a sister clade with *P. abieticola, P. ligustricola*, and *P. telopeae*. However, *P. endophytica* (S317-1) differs from *P. abieticola* by 28 nucleotides (18/570 in ITS, 5/211 in *act*, and 5/861 in nrLSU), from *P. ligustricola* by 37 nucleotides (27/648 in ITS and 10/210 in *act*) and from *P. telopeae* by 43 nucleotides (10/570 in ITS, 8/211 in *act*, 22/210 in *tef1*, 2/623 in *gapdh*, and 1/882 in nrLSU). Morphologically, the conidia of *P. endophytica* (9.0–11.0 μm) are wider than those of *P. abieticola* (7.0–8.0 μm) (Wikee et al. 2013b), *P. ligustricola* (4.9–7.4 μm) (Motohashi et al. 2008b), and *P. telopeae* (8.0–9.5 μm) (Yip 1989). Furthermore, the apical appendages of *P. endophytica* (7.0–15.0 μm) are shorter than those of *P. abieticola* (20.0–25.0 μm) (Wikee et al. 2013b) and *P. telopeae* (20.0–26.0 μm) (Yip 1989), and longer than those of *P. ligustricola* (4.9–9.8 μm) (Motohashi et al. 2008b). *Phyllosticta endophytica* has the same host as *P. cryptomeriae* Kawam., *Phyllosticta cunninghamii* Allesch., and *P. concentrica* Sacc. *P. cunninghamii* does not have molecular data (Brittingham et al. 1978). The conidia of *P. endophytica* (12.5–16.0 × 9.0–11.0 μm) are longer than those of *P. cryptomeriae* (9 − 13.5 μm) (Petrini et al. 1991) and *P. cunninghamii* (2.0–3.0 μm) (Sydow 1897) and wider than those of *P. cunninghamii* (1.0 μm) (Sydow 1897) and *P. concentrica* (6.0–9.0 μm) (Wikee et al. 2013a). Therefore, *Phyllosticta endophytica* is proposed as a new species.

**Figure 1.**
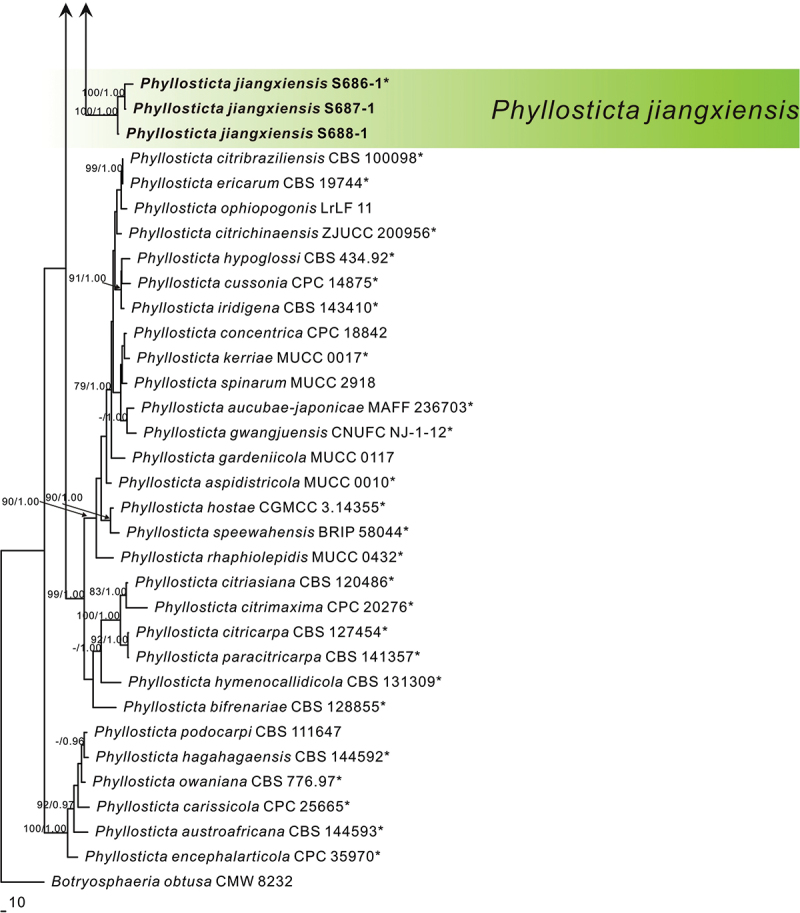
(Continued).

**Figure 3.**
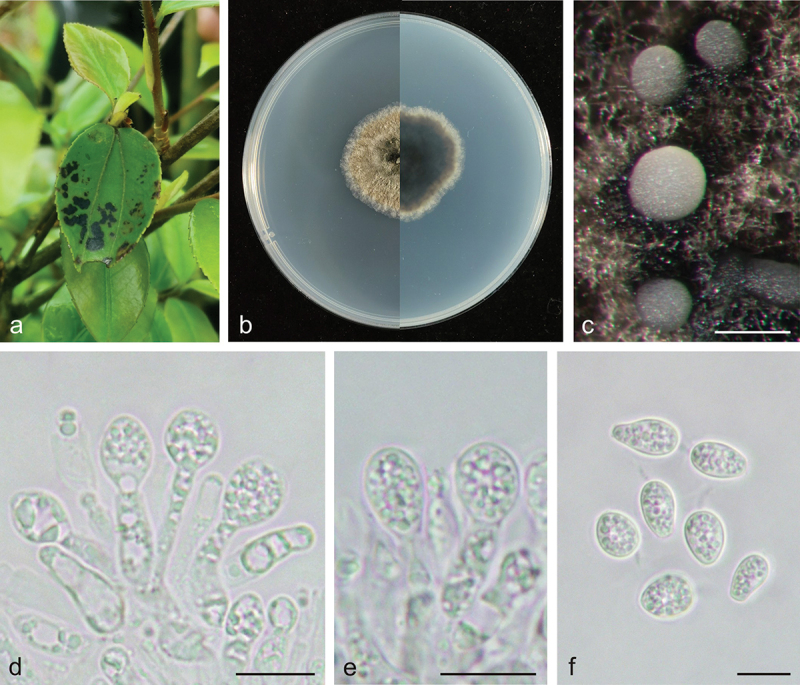
*Phyllosticta jiangxiensis* (holotype, CAF 8000202). a: Leaf lesions on living leaf of *Camellia oleifera*; b: Colonies (left-above, right-reverse) after 7 days on PDA; c: Conidiomata; d, e: Conidiogenous cells with conidia; f: Conidia. Scale bars: 200 μm (c); 10 μm (d–f).

***Phyllosticta jiangxiensis*** X.N. Sui & C.L. Hou, sp. nov. Figure 3

*MycoBank*: 848950.

 *Etymology*: *jiangxiensis*, derived from the province name of the type locality of this species, Jiangxi Province.

 *Type*: China, Jiangxi Province: Changbu Forest Plantation, on diseased leaves of *Camellia oleifera*, 22 April 2021, Q.T. Wang & C.L. Hou (holotype CAF 8000202, ex-holotype living culture CNUCC S686-1 = CFCC 59083).

 *Description*: Leaf spots irregularly circular, black (#000000), dark red (#270000) on the outer edge. Conidiomata not observed on leaves. Conidiophores are cylindrical to ampulliform, often reduced to conidiogenous cells. Conidiogenous cells 7.5–15.0 × 2.0–5.5 μm, hyaline, and cylindrical. Conidia 8.0–12.0 × 6.0–8.0 μm, mean ± SD = 9.7 ± 0.9 × 7.1 ± 0.4 μm, hyaline, aseptate, ovoid, ellipsoidal to subglobose, and surrounded by a thick mucoidsheath. Some conidia have thin and long apical appendages 4.0–14.0 μm.

 *Culture characteristics*: Colonies up to 4.3 cm in diameter at 7 days on PDA. Round with ciliate at the edge, dark lime green (#1f2820) to dark yellow (#8e7f00) on the obverse, greyish-white (#ececec) on the outer edge, and dark lime green (#171c1a) to light grey (#ececec) on the reverse side. Conidiomata are visible after 14 days with pale yellow (mostly white) (#ffffec) oozing on PDA.

 *Additional specimens examined*: China, Jiangxi Province: Changbu Forest Plantation, on diseased leaves of *Camellia oleifera*, 22 April 2021, Q.T. Wang & C.L. Hou, living culture S687-1. China, Jiangxi Province: Changbu Forest Plantation, on diseased leaves of *Camellia oleifera*, 22 April 2021, Q.T. Wang & C.L. Hou, living culture S688-1.

 *Notes*: In the phylogenetic analysis, the sequences of S686-1, S687-1, and S688-1 formed an independent clade with high support values in the multi-gene phylogenetic tree (MPB = 100, PP = 1.00, Figure 1). S686-1 was isolated from the leaves of *Camellia oleifera*, one of China’s four major woody oil plants. *Phyllosticta camelliae* Westend., *P. camelliaecola* Brunaud, *P. capitalensis, P. erratica* Ellis & Everh., *P. theae* Speschnew, and *P. theacearum* Aa have been reported on *Camellia oleifera*. Among these, only *P. capitalensis* and *P.* *camelliae* (Motohashi et al. 2009) have molecular data but are distantly related to *P. jiangxiensis* in the phylogenetic tree. Other species without molecular data can be distinguished by morphological characters. For example, *P. jiangxiensis* produces bigger conidia (8.0–12.0 × 6.0–8.0 μm) than *P. camelliaecola* (5.0–6.0 × 2.0–3.0 μm) (Brunaud 1888), *P. theae* (6.0–8.0 × 1.5–2.0 μm) (Speschnew 1904), and longer conidia than *P. erratica* (6.0–8.0 μm) (Ellis and Everhart 1900). Furthermore, *P. jiangxiensis* have longer conidiogenous cells (7.5–15.0 × 2.0–5.5 μm) than *P. theacearum* (4.0–6.0 × 2.0–2.5 µm) (Van der Aa Ha 1973), *P. capitalensis* (7.0–10.0 × 3.0–5.0 μm) (Glienke et al. 2011). Therefore, *Phyllosticta jiangxiensis* is proposed as a new species.

**Figure 4.**
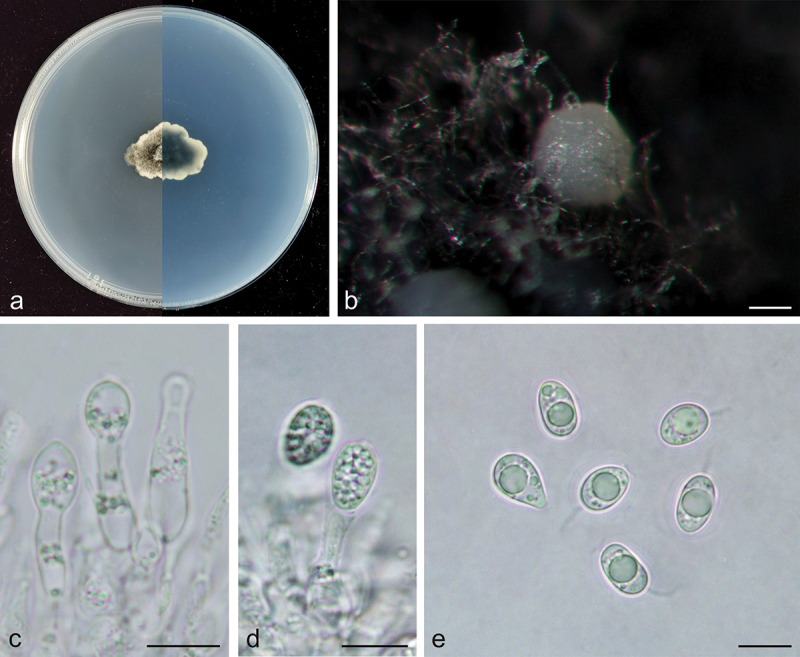
*Phyllosticta machili* (holotype, CAF 8000203). a: Colonies (left-above, right-reverse) after 7 days on PDA; b: Conidiomata; c, d: Conidiogenous cells with conidia; e: Conidia. Scale bars: 200 μm (b); 10 μm (c–e).

***Phyllosticta machili*** X.N. Sui & C.L. Hou, sp. nov. Figure 4

*MycoBank*: 848951.

 *Etymology*: *machili*, from the genus name of the host plant, *Machilus*.

 *Type*: China, Jiangxi Province: Xinyu Fir Plantation, on diseased leaves of *Machilus pauhoi*, 22 April 2021, Q.T. Wang & C.L. Hou (holotype CAF 8000203, ex-holotype living culture CNUCC S661-1 = CFCC 59084).

 *Description*: Conidiogenous cells 12.0–22.0 × 2.5–4 μm, hyaline, and cylindrical. Conidia 9.0–13.5 × 6.5–9.0 μm, mean ± SD = 11.0 ± 1.1 × 8.0 ± 0.7 μm, hyaline, aseptate, ovoid, ellipsoidal to subglobose, containing a single large central guttule, and surrounded by a thick mucoid sheath. Some conidia have apical appendages 3.0–7.0 μm.

 *Culture characteristics*: Colonies up to 2.2 cm in diameter at 7 days on PDA. The entire edge is irregular, dark greyish tallow colour (#4a4a36) with aerial hyphae to greyish-white (#e0e0e0) on the obverse, dark greyish lime green (#2a392a) on the outer edge, and dark greyish lime green (#2a392a) to greyish-white (#e0e0e0) on the reverse side. Conidiomata are light grey (#c9c9c9) and visible after 14 days.

 *Additional specimens examined*: China, Jiangxi Province: Xinyu Fir Plantation, on diseased leaves of *Machilus pauhoi*, 22 April 2021, Q.T. Wang & C.L. Hou, living culture S662-1.

 *Notes*: In the phylogenetic analysis, two strains of *P. machili* clustered in a single clade with high support values (MPB = 98, PP = 1.00, Figure 1), and *P. machili* was closely related to *P. ardisiicola, P. capitalensis*, and *P. pterospermi* (MPB = 74, PP = 0.97, Figure 1). However, *P. machili* (S661-1) differs from *P. capitalensis* by 29 nucleotides (13/565 in ITS, 13/232 in *act*, 3/214 *tef1*, and 9/639 in *gapdh*), from *P. pterospermi* by 38 nucleotides (14/617 in ITS, 12/250 in *act*, 3/380 in *tef1*, 3/835 in *gapdh*, and 3/882 in nrLSU), and from *P. ardisiicola* by 34 nucleotides (23/625 in ITS and 11/214 in *act*). Morphologically, *P. machili* can be distinguished by producing longer conidiogenous cells (12.0–22.0 × 2.5–4 μm) than *P. pterospermi* (7.5–11.0 × 2.5–4.5 μm) (Zhang et al. 2022), *P. capitalensis* (7.0–10.0 × 3.0–5.0 μm) (Glienke et al. 2011), and *P. ardisiicola* (5–12.5 × 1.2–2.5 μm) (Motohashi et al. 2008b). *Phyllosticta plumbaginicola* V.G. Rao (8–13 μm) (Nag Raj 1993) does not have molecular data but has similar morphological characters to *P. machili*; however, *P. machili* has shorter apical appendages (3–7 μm). Combined with phylogenetic analysis and morphological comparisons, this fungus was identified as a new species. *Phyllosticta machili* is the first *Phyllosticta* species reported from *Machilus*.

**Figure 5.**
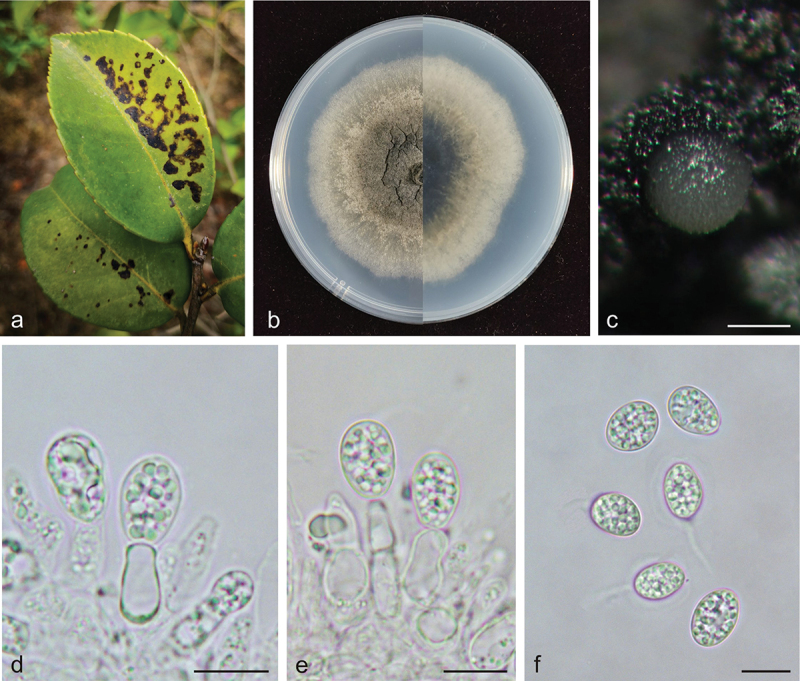
*Phyllosticta xinyuensis* (holotype, CAF 8000204). a: Leaf lesions on living leaf of *Camellia oleifera*; b: Colonies (left-above, right-reverse) after 7 days on PDA; c: Conidiomata; d, e: Conidiogenous cells with conidia; f: Conidia. Scale bars: 200 μm (c); 10 μm (d–f).

***Phyllosticta xinyuensis*** X.N. Sui & C.L. Hou, sp. nov. Figure 5

*MycoBank*: 848952.

 *Etymology*: *xinyuensis*, derived from the type locality of this species, Xinyu City.

 *Type*: China, Jiangxi Province: Changbu Forest Plantation, on diseased leaves of *Camellia oleifera*, 22 April 2021, Q.T. Wang & C.L. Hou (holotype, CAF 8000204, ex-holotype living culture CNUCC S668-1 = CFCC 59085).

 *Description*: Leaf spots irregularly circular, black (#000000), dark red (#270000) on the outer edge. Conidiomata not observed on leaves. Conidiophores are cylindrical to ampulliform, often reduced to conidiogenous cells. Conidiogenous cells 9.0–16.0 × 3.0–5.0 μm, hyaline, and cylindrical. Conidia 10.0–14.0 × 7.0–10.0 μm, mean ± SD = 12.2 ± 0.9 × 8.0 ± 0.7 μm, hyaline, aseptate, ovoid, ellipsoidal to subglobose, and surrounded by a thick mucoid sheath. Some conidia have thin and long apical appendages 3.0–7.0 μm.

 *Culture characteristics*: Colonies up to 6.9 cm in diameter at 7 days on PDA. Round with a scalloped margin, dark greyish-green centre (#5a6053) to greyish-green edges (#808976) on the obverse and reverse sides. Formation of olive green to green lamellae as black masses. Conidiomata are visible after 12 days with light grey (mostly white) (#f8f8f8) tendrils on PDA.

 *Additional specimens examined*: China, Jiangxi Province: Changbu Forest Plantation, on diseased leaves of *Camellia oleifera*, 22 April 2021, Q.T. Wang & C.L. Hou, living culture S669-1.

 *Notes*: In the phylogenetic analysis, the two strains of *P. xinyuensis* clustered together in a clade with high support values (MPB = 100, PP = 1.00, Figure 1) and grouped with *P. beaumarisii* A.P. Paul & M.D. Blackburn. Morphologically, *P. xinyuensis* produces wider conidia (10.0–14.0 × 7.0–10.0 μm) than *P. beaumarisii* (7.5–15 × 6.5–8.75 μm) (Paul and Blackburn 1986). *Phyllsoticta camelliae, P. camelliaecola, P. capitalensis, P. erratica, P. theae*, and *P. theacearum* have the same host as *P. xinyuensis*. Among these, only *P. capitalensis* and *P. camelliae* (Motohashi et al. 2009) have molecular data but are distantly related to *P. xinyuensis* in the phylogenetic tree. Other species without molecular data can be distinguished by morphological characters. For example, *P. xinyuensis* produces wider conidia (10.0–14.0 × 7.0–10.0 μm) than *P. camelliaecola* (5.0–6.0 × 2.0–3.0 μm) (Brunaud 1888), *P. theae* (6.0–8.0 × 1.5–2.0 μm) (Speschnew 1904), *P. theacearum* (8.0–12.5 × 5.5–7.0 μm) (Van der Aa Ha 1973), *P. capitalensis* (11.0–12.0 × 6.0–7.0 µm) (Glienke et al. 2011) and longer conidia than *P. erratica* (6.0–8.0 μm long) (Ellis and Everhart 1900). Therefore, *Phyllosticta xinyuensis* is introduced as a novel species.

## 4. Discussion

The initial identification of *Phyllosticta* species was mainly based on morphology, culture characteristics, and host associations. Van der Aa and Vanev (2002) revised more than 2,000 species in *Phyllosticta* and suggested more accurate and reliable morphological characteristics for its classification. However, the *Phyllosticta* species share many morphological similarities, making it difficult to classify them accurately using morphology alone. Hyde et al. (2010) proposed a connection between molecular data and the correct taxonomic unit using morphological characters in combination with molecular data to accurately identify fungi. Wikee et al. (2012) placed *Phyllosticta* within *Phyllostictaceae* using molecular phylogenetic analysis based on the ITS, nrLSU, *act, tef1*, and *gapdh* genes, combined with morphological characters.

In this study, four new species were described based on morphology and phylogenetic analysis. Single-gene phylogenetic trees for each of the five genes were also constructed separately. However, the multi-locus phylogenetic tree provided better resolution for the *Phyllosticta* species. Including protein-coding genes in the analysis greatly facilitated species-level identification, also demonstrated by Wikee et al. (2011). Multi-locus phylogeny and three signal locus (ITS, *tef1*, and *act*) supported the four new species. The difference between the topology of the single-gene phylogenetic trees of nrLSU and *gapdh* with that of the multi-locus phylogenetic tree may be due to fewer available sequences (only 32 linked data for *gapdh* in 71 recognised species, and 34 linked data for *gapdh* in 71 recognised species). Among the phylogenetic trees for a single locus, the *gapdh* gene tree showed the greatest difference in topological structure compared with the multi-gene phylogenetic tree. This difference may be due to the few available genes for *gapdh* (only 37 linked data for *gapdh* in 70 recognised species).

*Phyllosticta* species can cause lesions on many plant species with a wide host range. The 10 strains in this study were collected from three host species, including *Camellia oleifera, Machilus pauhoi*, and *Cunninghamia lanceolata*. Among these strains, *P. machili* is the first reported isolation from *M. pauhoi*; whether the species is host-specific needs to be determined by pathogenicity testing. Until now, six species of *Phyllosticta* isolated from *C. oleifera* have been reported, i.e. *P. camelliae, P. camelliaecola, P. capitalensis, P. erratica, P. theae*, and *P. theacearum*. Among these species, *P. capitalensis* has a wide host range, with nearly 357 species, whereas the other species have been isolated only from *C. oleifera* (Norphanphoun et al. 2020). *Phyllosticta cryptomeriae, P. concentrica*, and *P. cunninghamii* have previously been isolated from *Cu. lanceolata*. In addition, *Phyllosticta cunninghamii* has also been isolated from *Rhododendron cinnamomeum* var. *cunninghamii* Paxton (Norphanphoun et al. 2020). *P. cryptomeriae* can be parasitic on *Cryptomeria japonica* (L.f.) D. Don. and *P. concentrica* has a wide range of hosts, with about 40 host species. Therefore, species of *Phyllosticta* may not have host specificity for *C. oleifera* and *Cu. lanceolata*. In addition, host specialisation in *Phyllosticta* may be related to lifestyle, and the endophytic fungus is less host-specific than the pathogenic fungus (Wikee et al. 2011); thus, *P. capitalensis* has a wider host range, which may be related to its endophytic lifestyle. A more in-depth study of whether there is host specificity and how it relates to fungal lifestyle would require a comparative analysis of genomic data, such as comparing the number and type of carbohydrate-active enzymes.

According to Okane et al. (2003), *Phyllosticta* species, including pathogens, latent pathogens, and endophytes, are widely distributed and have a variety of lifestyles. *Phyllosticta endophytica* was isolated as an endophytic fungus from *Cu. lanceolata* in this study. Endophytes can transform into pathogenic organisms under certain circumstances (Rodriguez and Redman 2008; Rodriguez et al. 2009). Therefore, whether these species have specific hosts, their habitats, lifestyles, and the conditions under which they may transform need further investigation. This has important implications for the potential biological control of plant diseases.
